# From science to real-life oncology—the ECCO 2018 European Cancer Summit, 7–9 September 2018, Vienna, Austria

**DOI:** 10.3332/ecancer.2018.877

**Published:** 2018-10-15

**Authors:** Sarah Liptrott

**Affiliations:** IRCCS, European Institute of Oncology, Milan, Italy

**Keywords:** cancer care, efficiency, quality, Europe, policy development

## Abstract

The European CanCer Organisation (ECCO) held its first European Cancer Summit in Vienna, Austria, from 7–9 September 2018. The summit, ‘From Science to Real-life Oncology’, attracted over 370 participants from across the globe. The aim of the event was connecting science with real life through policy evolution. A broad range of stakeholders attended the event including representatives of ECCO Member Societies, healthcare professionals, pharmaceutical company representatives, patient organisations including patient advocates, commercial providers to the healthcare sector, EU and national/regional government officials as well as academic researchers and regulatory professionals. The summit provided the opportunity to discuss some challenging issues including a European agenda on cancer, the use of big data and putting a price on cancer medicines. One of the objectives of the summit was to gather stakeholder decisions on resolutions looking at efficiency in cancer care, integration of services and quality. The result was a highly interactive well-attended meeting which permitted networking opportunities across stakeholder groups and giving direction to European cancer care.

The summit began with expert perspectives on future policies in cancer across Europe and an opening address from Her Excellency Marie-Loiuise Coleiro Preca, the President of Malta. Dr Vytenis Andriukaitis, the EU Commissioner for Health & Food Safety highlighted how co-operation and solidarity were the cornerstones to collaboration in cancer care and research for Europe and beyond. Raising awareness of issues in relation to cancer was felt to be a priority not only for commissioners but also for all citizens, as there was recognition that across member states in the EU, cancer strategies were not comparable. Dr Dinah Singer from the National Institute of Health (US) presented the Cancer Moonshot a program to accelerate efforts to prevent, diagnose, and treat cancer and the resulting initiatives that have been developed since 2017. This was followed by a discussion of the Cancer Groundshot concept by Professor Mark Lawler with the aim of public health benefit on global cancer outcomes.

The second session of the day, chaired by Professor Richard Sullivan from King’s College London (UK), looked at outcomes research, discussions beginning with ‘what are outcomes?’. Dr Claudia Allemani from the London School of Hygiene and Tropical Medicine (UK) presented data from CONCORD-3, the global programme for worldwide surveillance of cancer survival trends, which has highlighted increasing trends in breast cancer survival from 1995 to 2014, but still large differences in survival worldwide for women diagnosed in 2010–14. Results are rather different for lethal cancers such as lung, liver or pancreatic cancers, for which the prognosis in over 70 countries is still very poor [1]. These results come from ‘real-world’ big data for more than 37 million individual cancer patients collected by more than 320 population-based cancer registries. Dr John O’Donnell Global VP, Health Economics and Outcomes Research for Bristol-Myers Squibb (USA) described how traditional modelling techniques for outcomes may not truly reflect real-world data or what is happening to patients, especially where follow-up of patients is truncated. Dr Bettina Ryll from Melanoma Patient Network Europe evidenced how measuring outcomes does not automatically improve patient care, advocating for reflection on what outcomes are being measured and to whom these are important. Collaborative initiatives were encouraged such as CODE—the collaboration for oncology data in Europe and data sharing.

Finally, on the first day, the issue of big data was tackled including the new and evolving regulatory environment, and how citizens could be data donors. Professor Mark Lawler highlighted the example of patients with ROS1 mutations contacting each other online, sharing biospecimen and genomic data and taking this to the Bonnie J Addario Lung Cancer Foundation to move research and understanding forward. There was overall agreement that big data could be useful to identify gaps and issues allowing focus on areas for future research, but data collection needed to be standardised so as to facilitate an effective use of resources. There was undisputed agreement that patient involvement from the outset was fundamental to ensure collection of data addressing issues of importance to patients as well.

Day 2 began with looking at improving efficiency in cancer care. There was recognition of limited budgets, increasing numbers of patients and a lack of further resource availability. It was argued, however, that efficiency was not necessarily driven by money, but quality and teamwork were important components to be recognised in achieving efficiency. Professor Bengt Jӧnsson from the Stockholm School of Economics (Sweden) described economic models evaluating efficiency, however, financial and resource investment often did not provide comparable outcomes. Kathy Oliver from the International Brain Tumour Alliance went on to present patients perceptions of where inefficiency existed (initial results of the All Can study). The top three were the management of ongoing side effects, the psychological impact and at the point of cancer diagnosis. She also provided an example of how a simple and low-cost initiative provided efficient care—in this case—the use of a string of wool held by the child undergoing radiotherapy and the parent in the waiting room as a link, aiding anxiety management [2].

One of the key themes of the meeting was the development of resolutions on the organisation of cancer care. The first of the resolutions discussed was that of integration of cancer care and the essential requirements. Arguments here focused on the integration of primary care within cancer care. Dr Ian Banks, Chair of the ECCO Patient Advisory Committee asked the audience whether they felt primary care was a fully integrated part of cancer care and there was silence across the room. Only a handful of primary care representatives were present at the session. The significance of the primary care team as a key element was highlighted in a UK policy framework for cancer services over 20 years ago [3], yet true action in this direction was felt to be limited. The importance of primary care in the management of co-morbidities as well as psychological care was acknowledged although a presentation by Dr Tit Albreht highlighted the paucity of guidance for general practitioners for the care of cancer survivors. The session ended with voting on the principal resolution that by 2025, all national cancer plans in Europe should contain ambitious and measurable goals and actions to improve the integration of primary care healthcare professionals and informal carers within multidisciplinary care to patients. Voting was open to all members of the audience and presenters, which included healthcare professionals, patient advocates, commissioners, pharma representatives, academics and researchers and the resolution was well supported.

The second of the resolutions surrounded quality in cancer care and its measurement. Discussions initially began with that of ‘what is quality?’. There was agreement that while evidence-based medicine was one approach to quality and value, perceptions of these concepts may differ from the perspective of payers, commissioners or patients. The challenge is achieving agreement between stakeholders; however, suggestions included reflection on Patient Pathways and the use of quality indicators and patient-reported outcome measures to help in defining quality and value. The session ended with voting on the principal resolution that by 2023 an agreed set of core standards and evidence-based indicators (based on processes and patient outcomes) to measure the quality of all cancer services in European countries should be in place. Again voting was open to all members of the audience and presenters and over 90% supported the resolution.

Professor Richard Sullivan from Kings College London chaired the session on access and value. Professor Yolande Lievens, Past President of the European Society for Radiotherapy and Oncology referred to the ECCO position paper on access to innovation [4] and the importance of engaging professionals, patients and the care community to find sustainable solutions in cancer care. She highlighted work by the International Consortium for Health Outcomes Measurement (ICHOM) who aim to measure and report patient outcomes in a standardised way and have already produced Standard Set’s for health outcomes measurement for some of the more prevalent cancers. Dr Ajay Aggarwal from the Institute of Cancer Policy at Kings College London (UK) highlighted further considerations in the value debate including findings from his research where 50% of drugs entering the market had not demonstrated an improvement in length or quality of life [5]. So again, what is value? Professor Sullivan summarised, suggesting ‘value, like beauty, is in the eye of the beholder’.

The second part of this session was focused on value with contributions from nursing and psychologists perspectives. Dr Carole Farrell from the European Oncology Nursing Society evidenced the work of EONS with the RECaN Project and how nurses are contributing to patient outcomes. A meta-analysis following on from a literature review highlighted nurse-led interventions improving the quality of life globally as well as physical function, social and role function domains [6]. Dr Maria Die Trill, the President of the International Psycho-Oncology Society spoke about the ‘sixth vital sign’ and the evaluation of distress via the distress thermometer in clinical practice. She encouraged the involvement of psychosocial, behavioural and psycho-oncology scientists into global cancer treatment and prevention programs as well as integration in rehabilitation programs and as a routine part of cancer care.

The second day closed with the hot topic of ‘Putting a price on cancer medicines’. Eveline Scheres was the first speaker from the Association of European Cancer Leagues who advocate for cancer control and care at an EU level. She described the white paper due for publication in October this year on tackling the challenges in access to medicines for all cancer patients in Europe. Key points included the need for transparency in pricing and profits in order to promote trust between key stakeholders. Nathalie Moll, the Director General of the European Federation of Pharmaceutical Industries and Associates also agreed with transparency as a key component, however, she suggested that drug expenditure was only 20% of total expenditure so there were also existing healthcare system issues that also needed to be addressed. Furthermore, collaborative work needs to be undertaken to move towards access and agreeable solutions for all.

The third resolution was discussed on the final day of the summit: Survivorship and financial discrimination was the theme. Dr Marie Mesnil from the University of Rennes (France) spoke of the insurability of cancer survivors referring to French and Belgian approaches in legislation to address this, including the French law of ‘the right to be forgotten’ where cancer survivors are not obliged to declare their cancer after a defined period of time. It was recognised that new data surrounding survival—especially in the light of recent developments in immunotherapy treatments—were not being taken into account by insurance companies. Šarunas Narbutas from Youth Cancer Europe highlighted the challenge of getting leading insurance companies around the table to talks and the outdated mechanisms in place for calculating risk. Youth Cancer Europe will be holding an afternoon at the European Parliament on 17 October 2018 to discuss essential services for cancer survivors. Janette Rawlinson, a cancer survivor and patient advocate (UK) spoke of her experiences in relation to the occupational impact of cancer with particular focus on people who are self-employed and the unique challenges faced. She also highlighted the differences between cancers and stigma in the workplace, experiences mirrored in comments from the audience. The session ended with voting on the principal resolution that by 2025, in respect to accessing financial services, the right of cancer survivors not to declare their cancer 10 years after the end of the active treatment and 5 years if they had cancer under 18, should be codified across European countries. There was some debate regarding the term ‘active treatment’ and recognition of many patients who were in complete remission yet continued on treatment. It was deemed necessary to define this more clearly. Voting took place (with the note on active treatment) and was passed. The meeting then closed with a presentation from Professor Françoise Meunier from the European Organisation for Research and Treatment of Cancer delivering the Patrick Johnston Memorial Lecture.

## Conclusion

The summit highlighted a commitment to the provision of high-quality cancer care and this was a unique opportunity for so many different stakeholders to interact and network over the course of the 3 days. Many challenging issues were raised during the sessions and the variety of speakers sharing both complementary and sometimes contrasting perspectives permitted an interesting debate in which participants were well engaged. The agreed-upon resolutions determine ways to improve health systems, and ECCO and all key stakeholders have fundamental roles in realising the consensus of the agreement reached over the course of the summit.

## Conflicts of interest and funding

Sarah Jayne Liptrott is a member of the ECCO Patient Advisory Committee and her attendance at the meeting was reimbursed by ECCO.
